# Histone Deacetylase 1 Expression and Regulatory Network in Lung Adenocarcinoma Based on Data Mining and Implications for Targeted Treatment

**DOI:** 10.1155/2023/2745074

**Published:** 2023-01-04

**Authors:** Yueyuan Zhong, Mingdong Li, Shihui Guo, Minhua Li, Zitong Cao, Xueyao Luo, Jianhong Liu, Runzhang Liang, Yingqi Shao, Yue Yang, Xinqia Chen, Chuzhong Wei, Weijun Ling, Xiao Zhu, Yongmei Huang

**Affiliations:** ^1^The Marine Biomedical Research Institute, Guangdong Medical University, Zhanjiang, China; ^2^Department of Gastroenterology, Zibo Center Hospital, Zibo, China

## Abstract

**Background and Aims:**

Histone deacetylase 1 (HDAC1) codes a protein that is a component of the histone deacetylase complex. The abnormal expression of HDAC1 is strongly correlated with cell proliferation, differentiation, transcription, and translation. Through continuous screening of genes associated with changes in lung adenocarcinoma (LUAD), gene networks are formed to explore tumor pathogenesis and new therapeutic targets.

**Methods:**

We evaluated HDAC1 gene survival analysis and its expression of LUAD using relevant websites and databases (TCGA and GEO databases). Through data mining, we determined the frequency and type of HDAC1 mutation, obtained the relevant heat map of the gene interaction network, completed the analysis of gene ontology and function enrichment, and understood the pharmaceutic of HDAC1.

**Results:**

We found that HDAC1 expression was associated with the prognosis of patients with LUAD. In gene expression analysis, HDAC1 was highly expressed in LUAD, and the HDAC1 interaction gene network (MARCKSL, eIF3I) was closely related to cellular gene expression. Functional network analysis shows that the expression of HDAC1 is related to the monitoring point of the G1-S phase of the cell cycle and the activation of the Notch signaling pathway (CSL transcription factor), which is involved in the process of cell proliferation and differentiation and gene expression associated with new therapeutic targets.

**Conclusion:**

Our data revealed the expression and potential regulatory factors of HDAC1 in LUAD of data mining, which laid a foundation for the study of the occurrence, development, and treatment of HDAC1 in LUAD.

## 1. Introduction

Lung adenocarcinoma (LUAD) belongs to non-small-cell carcinoma, and it is the fastest-growing lung cancer incidence rate at present [1, 2]. Approximately 85% of lung cancer patients are diagnosed with non-small-cell lung cancer (NSCLC), which includes the histological subtypes of LUAD, squamous cell carcinoma, and large cell carcinoma. In the past, systemic cytotoxic chemotherapy has been the main treatment for advanced NSCLC, and new therapies are being developed. Currently, patients with LUAD are usually diagnosed using next-generation sequencing (NGS) for molecular testing to determine the best treatment for the patient. Epidermal growth factor receptor (EGFR) [3], BRAF-activated mutation, and anaplastic lymphoma kinase (ALK) [4] have been targeted as part of routine therapy [5–7]. At the same time, the success of immunotherapy has created a new paradigm of personalized therapy [8, 9], extended the survival rate of oncogene-driven patients with advanced LUAD, and accelerated the development of new drugs for LUAD, but success remains limited. The pathogenesis of LUAD is the interaction of polygenes and external factors, which is a complex pathological process of long-term development and formation. By continually screening genes for tumor-related changes to form gene networks, it may be possible to find out how tumors develop.

Histone deacetylase 1 (HDAC1) is known to be a component of the histone deacetylase complex. HDAC1 not only catalyzes histone acetylation and deacetylation in multisubunit complex but also interacts with retinoblastoma tumor suppressive proteins [10–12], which is a key factor in regulating eukaryotic gene expression control and cell proliferation and differentiation. Recent studies have shown that HDAC1 is involved in the pathogenesis of many cancers. Related studies have shown that HDAC1 gene expression is highly expressed in LUAD tissues [13, 14], and the related pathway affected by HDAC1 is closely related to the formation of cancer tissues.

At present, the related drugs targeting HDAC1 are mainly HDAC inhibitors [15, 16]. Natural synthetic histone deacetylase inhibitors (HDACI) are compounds with different target specificity and activity, mainly divided into four categories, including cyclic peptides, benzamide, short-chain fatty acids, and isohydroxamic acid [17]. HDACI induces different phenotypes in various transformed cells, including growth arrest, activation of apoptotic pathways, and autophagy death. All HDAC inhibitors can induce histone H3 hyperacetylation, which is related to the inhibition of proliferation, induction of cell differentiation, and apoptosis. Meanwhile, normal tissue cells have relatively greater resistance to HDACI-induced cell death [18, 19]. HDACI has been established as a new approach for the treatment of solid and hematologic tumors. By revealing the pathway of the role of HDAC1 in LUAD tissue, we may have the opportunity to find new targets and strategies for the diagnosis and treatment of LUAD, making the treatment of LUAD more accurate and effective.

## 2. Methods

### 2.1. Survival Analyses of HDAC1

Sources of the Kaplan–Meier plotter database include Gene Expression Omnibus (GEO), The European Genome-phenome Archive (EGA), and The Cancer Genome Atlas (TCGA) [20]. The tool enables univariate and multivariate Cox proportional hazard survival analyses using data generated from genomic, transcriptomic, proteomic, or metabolomic studies. We completed the survival analysis of HDAC1 in LUAD patients using this platform [21]. In statistical analysis, if the *P* value was less than 0.05, it was considered that the expression of HDAC1 was related to the prognosis of patients.

### 2.2. Oncomine Analysis

Oncomine (https://www.oncomine.org/) is currently the world's largest oncogene chip database and integrated data-mining platform [22]. The database collected data from 729 gene expression datasets and more than 90,000 cancers and normal tissue samples. Differential expression analysis was performed by *t*-test as a measure of differential expression, and the false discovery rate was calculated to correct for significance. HDAC1 copies the number of DNA in Oncomine calls to TCGA database data completion [23]. The mRNA differential expression analysis was performed on the data of the gene expression profile chip in the GEO database called by Oncomine. The analysis studied a range of LUAD, including Weiss Lung, Okayama Lung, Hou Lung, and Beer Lung. We analyzed the expression of HDAC1 in LUAD tissues and normal tissues. If the fold change is less than 2, there is no significant differential expression of the gene, and the *t* value in statistical analysis is less than 0.05, and it is considered that there is a differential expression in LUAD tissues and normal tissues, and the lower the *P* value, the higher the degree of difference.

### 2.3. UALCAN Analysis

UALCAN (https://ualcan.path.uab.edu/index.html) is an effective cancer data online analysis and mining based on the TCGA database website [24]. The website analyzes the relative gene expression of tumors, normal samples, and tumor subgroups using a *t*-test. In the process of the study, we used the UALCAN website to call the mRNA sequencing data in the TCGA database to carry out the comparative analysis of different ages, sexes, races, smoking or not, disease stages, tumor grades, and TP53 lesions with normal tissues and to verify the mRNA differential expression in the previous step. At the same time, the HDAC1 protein expression profile was analyzed, and the expression of HDAC1 gene protein at the total protein level, phosphorylated protein level, and pan-cancer level could be obtained. Through Pearson correlation analysis on this website, we completed gene correlation analysis of HDAC1 and obtained gene sets interacting with target genes, to facilitate subsequent mRNA expression analysis of gene sets and molecular network analysis of interaction.

### 2.4. C—BioPortal Analysis

C—BioPortal (https://www.cbioportal.org) is a powerful TCGA analysis platform that integrates the genomic data of 164 cancers such as TCGA, ICGC, and GEO [25, 26]. We analyzed the mutation profile of the HDAC1 gene in LUAD samples to analyze mutation levels to analyze how the gene functions in cancerous tissues.

### 2.5. LinkedOmics Analysis

LinkedOmics is a publicly available portal that includes multiomics data from 32 TCGA cancer types [27]. It also includes MS-based proteomics data generated by the Clinical Proteomic Tumor Analysis Consortium (CPTAC). Thus, heat maps of mRNA expression of some genes are positively associated with HDAC1, and those negatively associated with HDAC1 in LUAD were obtained.

### 2.6. STRING Analysis

The STRING database is an online database for searching known protein interaction relationships [28], which stores 2031 species, 9643763 proteins, and 1380838440 interaction information. In our work, we input the previously obtained gene sets related to HDAC1, to obtain the visual interaction relationship of gene sets, and so as to understand our target genes more intuitively.

### 2.7. WEBGESTAIT Analysis

WEBGESTAIT enrichment analysis (https://www.webgestalt.org/option.php) is a focus of the online website [29, 30], supports multiple enrichment analysis methods, and covers the functional annotation database comprehensively. We used this website to obtain the enrichment results of HDAC1 and its related gene sets, which intuitively showed that genes were related to those pathways in Gene Ontology (GO), to explore and verify the functions of genes.

### 2.8. KOBAS (KEGG Orthology Based Annotation System) Analysis

KOBAS 3.0 (https://kobas.cbi.pku.edu.cn/kobas3) is widely used in gene and protein function annotation databases [31]. We analyzed the Kyoto Encyclopedia of Genes and Genomes (KEGG) via the website. After entering related search options, we found relevant KEGG functional pathways. We investigated the possible role of HDAC1 in cancer tissue formation.

### 2.9. DRUGSURV Analysis

DRUGSURV (https://www.bioprofiling.de/GEO/DRUGSURV/) is a tumor drug database for querying and analyzing genes related to clinical prognosis [32, 33]. The database collects about 1700 FDA-approved drugs. The database contains 5000 investigational drugs and related target genes, as well as 17 associated tumors and about 50 clinical prognostic data. Through this website, we can check the latest progress of HDAC1 drug development and related target genes.

## 3. Results

### 3.1. Gene Survival Analysis of HDAC1

**Figure 1:**
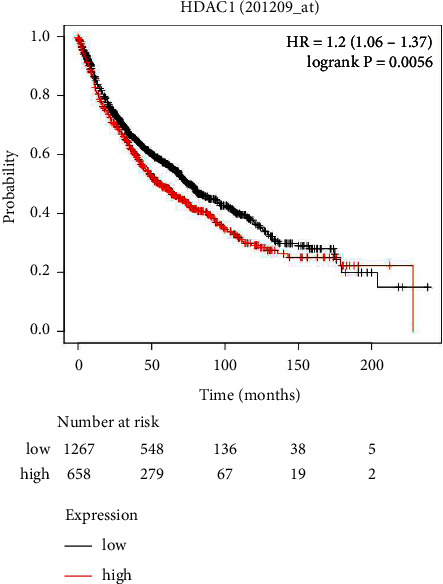
Survival analysis of HDAC1 expression of LUAD patients.

We obtained the survival analysis of HDAC1 for LUAD patients using the Kaplan–Meier plotter platform (Figure 1). The results showed that there was a statistically significant difference in survival between patients with high HDAC1 expression and patients with low HDAC1 expression (hazard rate: HR = 1.2, log-rank*P*=0.0056). This suggests that high expression of this gene is a risk factor leading to increased mortality.

### 3.2. Expression of HDAC1 in LUAD

**Figure 2:**
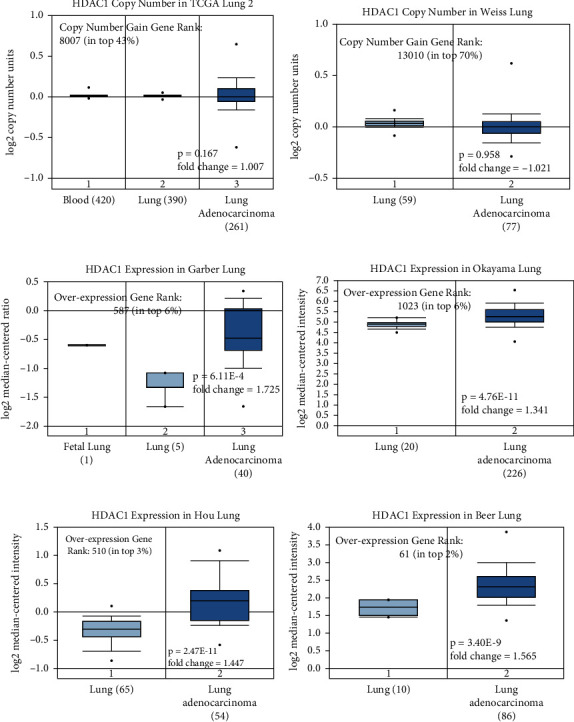
DNA copy number and mRNA levels of HDAC1 in LUAD. The DNA copy number data were not statistically significant, while the mRNA expression level was significantly higher than that of normal tissue, according to Oncomine analysis, showing fold change, associated *P* values, and the ranking of overexpressed genes. The (a)–(b) figures show the copy number of HDAC1 in the tumor genome atlas (TCGA) lung 2 and Weiss Lung datasets, respectively. The (c)–(f) figures show the data of the gene expression chip in the GEO database of HDAC1 mRNA levels in Okayama Lung, Garber Lung, Hou Lung, and Beer Lung, respectively.

**Figure 3:**
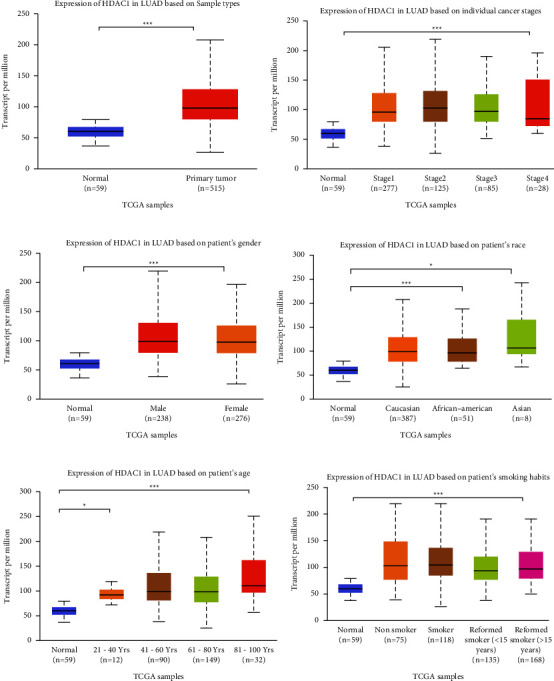
HDAC1 mRNA expression obtained by mRNA sequencing. (a) Boxplot of HDAC1 relative expression in normal tissue samples and LUAD samples. (b) Relative expression histogram of HDAC1 in normal tissue samples and in different LUAD grades. (c) Boxplot of HDAC1 mRNA expression in Caucasians, African Americans, and Asian ethnic groups compared with normal samples. (d) Comparison of mRNA levels of HDAC1 normally expressed in individuals with mRNA levels expressed in male and female LUAD patients. (e) Comparison of LUAD expression between normal individuals and LUAD patients aged 21–40, 41–60, 61–80, and 81–100 years. (f) Comparison of LUAD expression between normal individuals and nonsmokers, smokers, smokers for less than 15 years, and smokers for more than 15 years. ^*∗*^, *P* < 0.05; ^*∗∗*^, *P* < 0.01; ^*∗∗∗*^, *P* < 0.001; n.s, no significant. All the data in the above figures were statistically significant.

**Figure 4:**
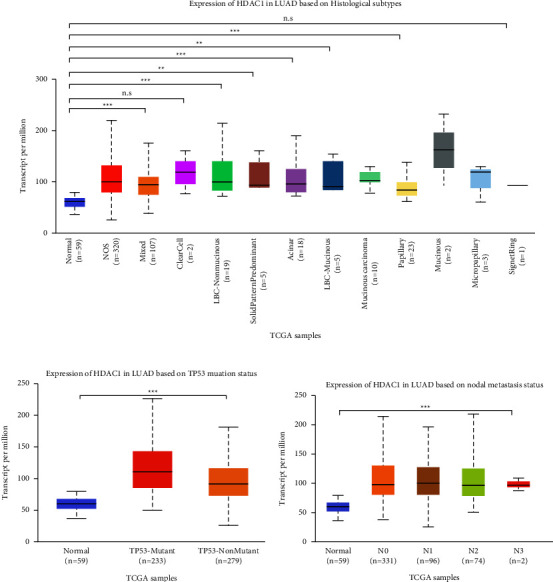
HDAC1 mRNA expression obtained by mRNA sequencing method. (a) Comparison of HDAC1 mRNA levels in normal individuals and in various histological subtypes (NOS, mixed, clearcell, LBC-nonmucinous, etc.). (b) Comparison of HDAC1 mRNA levels in normal individuals and in different lymph node metastatic states. (c) Comparison of HDAC1 mRNA expression levels in normal individuals and TP53 mutated and TP53 unmutated conditions. ^*∗*^, *P* < 0.05; ^*∗∗*^, *P* < 0.01; ^*∗∗∗*^, *P* < 0.001; n.s: no significant.

**Figure 5:**
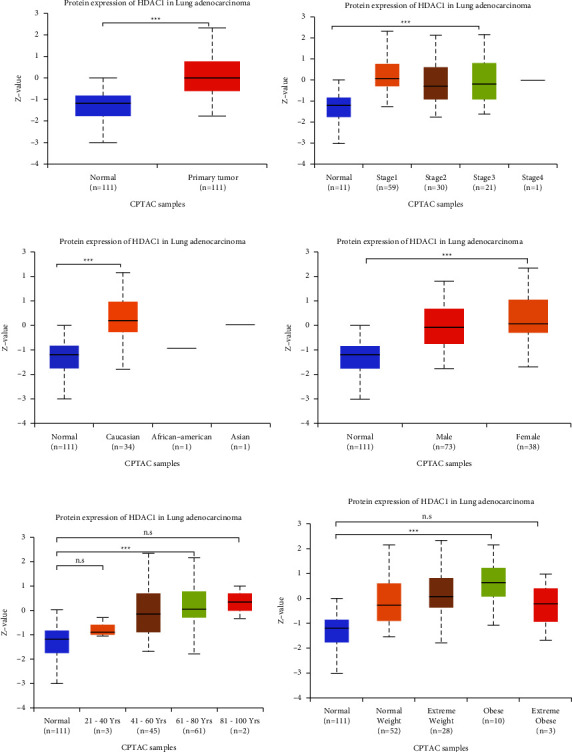
HDAC1 protein expression level in LUAD was analyzed by UALCAN. (a) Statistical comparison of total protein expression levels of HDAC1 in normal individuals and patients with LUAD. (b) The total protein expression level of HDAC1 in normal individuals and patients with different stages of LUAD. (c) The total protein expression levels of HDAC1 in normal individuals, white people, African Americans, and Asian people. (d) Comparison of the total protein expression levels of HDAC1 in normal individuals and LUAD patients of different genders. (e) Comparison of the total protein expression levels of HDAC1 in LUAD patients of different ages and normal individuals. (f) Expression of HDAC1 in total protein levels at different levels of obesity. ^*∗*^, *P* < 0.05; ^*∗∗*^, *P* < 0.01; ^*∗∗∗*^, *P* < 0.001; n.s: no significant.

**Figure 6:**
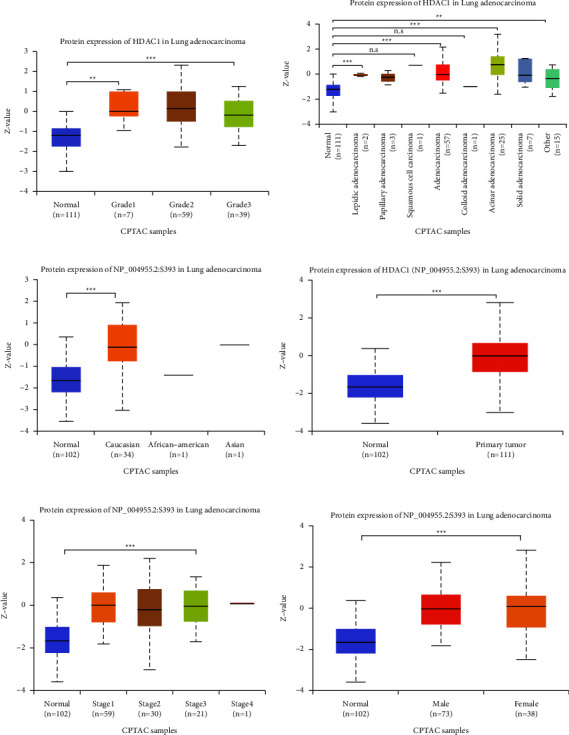
The differences in HDAC1 total protein expression level and phosphorylated protein expression level in LUAD patients under different conditions were obtained by UALCAN database analysis. (a) Comparison of total protein expression levels of HDAC1 in different tumor grades in LUAD with those in normal individuals. (b) Comparison of total protein expression levels of HDAC1 in different tissue adenocarcinoma tissues and normal individuals. (c) HDAC1 phosphorylated protein expression levels in white, African American, and Asian patients with LUAD. (d) Comparison of HDAC1 phosphorylated protein expression levels in LUAD patients and normal individuals. (e)–(f) Comparison of HDAC1 phosphorylated protein expression levels in normal individuals with different LUAD grades and different sexes. ^*∗*^, *P* < 0.05; ^*∗∗*^, *P* < 0.01; ^*∗∗∗*^, *P* < 0.001; n.s: no significant.

**Figure 7:**
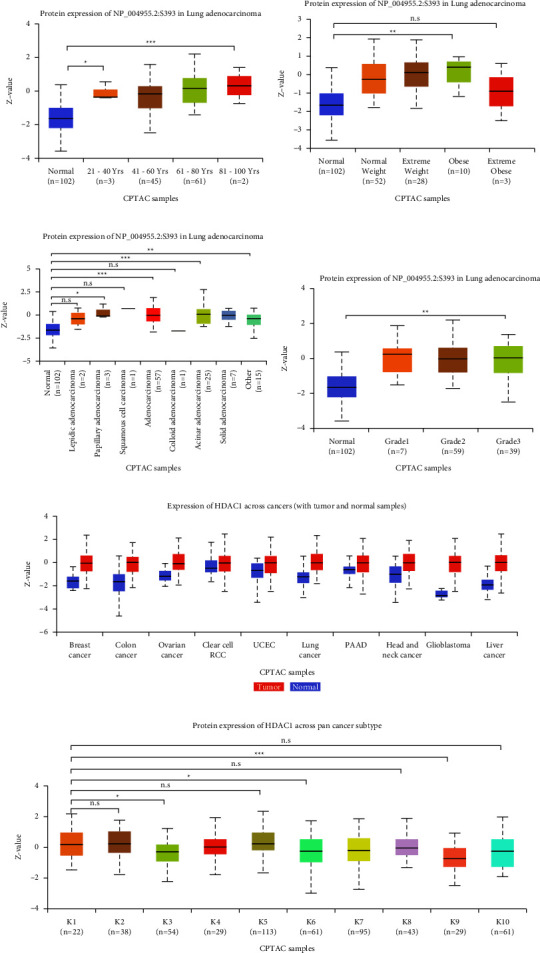
Expression levels of HDAC1 phosphorylated proteins in different LUAD patients and different cancers. (a)–(c) Expression levels of HDAC1 phosphorylated proteins in LUAD patients with different ages (21–40 years old, 41–60 years old, 61–80 years old, 81–100 years old), different obesity levels, and different tumor grades. (d) The expression level of HDAC1 phosphorylated protein in different adenocarcinoma tissues. (e) Expression of HDAC1 in different cancers. (f) Expression of HDAC1 phosphorylated proteins in different pan-carcinomatous subtypes (*K*1–*K*10). ^*∗*^, *P* < 0.05; ^*∗∗*^, *P* < 0.01; ^*∗∗∗*^, *P* < 0.001; n.s: no significant.

We analyzed and evaluated the copy number variation (CNV) analysis of DNA of the target gene HDAC1 and the chip difference analysis of HDAC1 mRNA gene expression profile through TCGA and GEO databases (Figures 2(a)–2(f)). There was no significant difference in gene copy number in cancer tissues compared with normal tissues (*P* > 0.05). HDAC1 mRNA expression in some LUAD tissues was significantly higher than that in normal tissues (*P* < 0.01). Although the data showed that the differential multiples of HDAC1 were all within 2, HDAC1 mRNA overexpression ranked in the top 2%–6% in some cancer tissues. Meanwhile, we further understood the mRNA expression of HDAC1 through the mRNA sequencing data in the TCGA database which can be called by the UALCAN database (Figures 3(a)–3(f)). We see, according to age, gender, race, smoking, disease stage, tumor grade, and TP53 lesions, whether the database HDAC1 in cancerous tissue translation level is significantly higher than that of normal tissue (Figures 4(a)–4(c)). HDAC1 gene expression increases sharply after TP53 mutation, and deacetylation of HDAC1 gene expression product may lead to the occurrence of cancer. At the same time, differential analysis of HDAC1 protein expression profile was performed to analyze the level of total protein and phosphorylated protein, as well as the expression of HDAC1 gene protein at the pan-carcinoma level (Figures 5–7). In the protein expression profile, HDAC1 expression in cancer tissues was higher than that in normal tissues in most of the protein expression categories. It can be seen that the mRNA expression of HDAC1 may be closely related to the pathogenesis of LUAD. Perhaps, we can explore the correlation between HDAC1 and LUAD as a diagnostic indicator or treatment plan.

### 3.3. Genomic Changes of HDAC1 in LUAD

**Figure 8:**
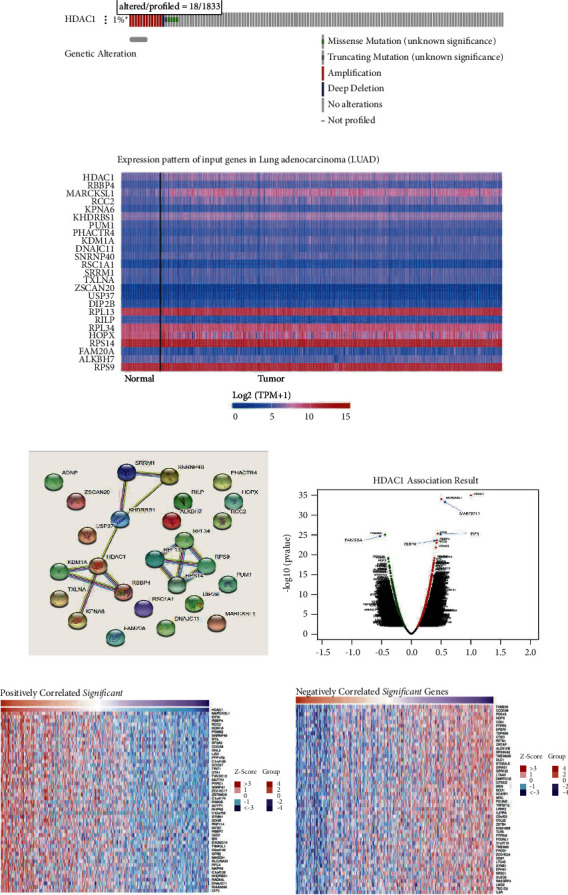
HDAC1 mRNA expression and molecular network analysis of DNA genetic changes and all genes in the related gene set at the cBioPortal website. (a) HDAC1 mutation frequency in LUAD, dark red represents amplification, blue represents deep silencing, green represents missense mutation (meaning unknown), and gray represents no mutation. (b) Heat map of transcriptional expression of this gene set. The data of mRNA expression of this gene set were obtained from LUAD tissue samples of TCGA. On the left is a normal control sample, and on the right is a tumor tissue sample, showing increased expression in red and decreased expression in blue. (c) 25 genes set network construction. (d) Correlation Pearson chart of gene set composed of 25 genes related to HDAC1. This chart represents a visual display of many genes related to the HDAC1 gene. The left side of the abaxial axis is a negative value (negative correlation), and the right side is a positive value (positive correlation). (e)–(f) Heat map of HDAC1 positive and negative associated gene sets. The left side of each graph is a positive correlation, and the right side is a negative correlation. In the graph, the darker the red is, the higher the correlation, while the darker the blue is, the lower the correlation.

We obtained the frequency and type of HDAC1 mutations through the cBioPortal database (Figure 8(a)). The results showed that 18 of 1833 individuals had HDAC1 mutations (1%), including missense mutations, deletion mutations, deep deletions, and amplifications. The most common type of DNA variation in HDAC1 is amplification.

### 3.4. Biological Interaction Gene Network of HDAC1 in LUAD

We obtained some genes that may interact with HDAC1 by TCGA analysis in UALCAN. By Pearson correlation (Person correlation coefficient, Person—CC), we chose 16 related genes (|*p*| ≥ 0.50) and 8 negatively related genes. The data in the figure are obtained by TCGA analysis, cBioPortal, STRING, and LinkedOmics analysis of 25 genes (Figures 8(b)–8(f)). HDAC1 was associated with KDM1A, RBBP4, KPNAB, KHDRBS1, SRRM1, and SNRNP40 in the gene interaction network. We can see that HDAC1 and macrophage myristylated alanine-rich C kinase (MARCKSL1) (positive rank #1, Pearson correlation = 0.61,*P* < 0.001), and HDAC1 and Eukaryotic Translation Initiation Factor 3 Subunit I (EIF3I) (Pearson correlation = 0.45, *P* < 0.001) had interaction.

### 3.5. Functional Enrichment of HDAC1 Gene

**Figure 9:**
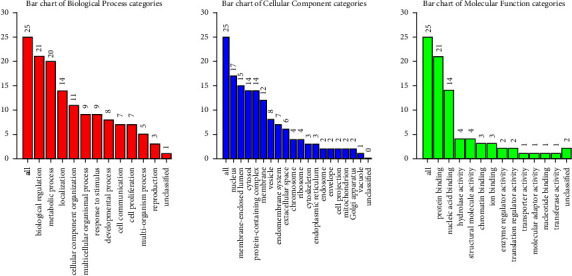
GO annotation enrichment analysis of 24 HDAC1-related genes. The number of HDAC1 genes in the three functional pathways of biological processes, cell components, and molecular functions was studied on the website.

**Figure 10:**
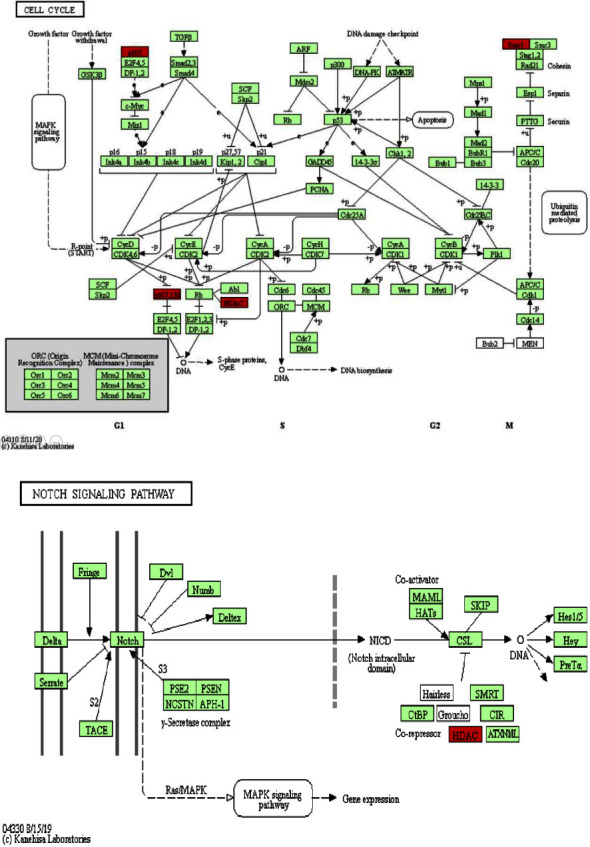
The KEGG pathway of HDAC1 was analyzed by KOABS. (a)–(b) showed that HDAC1 was involved in the KEGG cell cycle and Notch signaling pathways, respectively.

By comparing and analyzing 25 genes related to HDAC1 with the GO database (Figure 9), the results showed that 21 genes in biological process categories were involved in biological regulation. In the cellular component categories, 17 genes were concentrated in the nucleus. Among the molecular function categories, 21 genes were enriched in protein binding. We again analyzed the related expressions of 25 HDAC1-related genes in the KEGG pathway through the KOBAS website (Figure 10) to show the functional pathways in the cell cycle and related signaling pathways.

### 3.6. HDAC1 Drug Target

A list of targeted drugs targeting HDAC1 was obtained through the drug–gene interaction database (DGLDB). This list shows that drugs are mainly targeted to inhibit HDAC1 (Table S1). The main categories of drugs are hydroxylamine acids, short-chain fatty acids, amides, cyclotetrapeptides without epoxide groups, etc. At present, there are several HDAC inhibitors in the clinical research stage, which can effectively inhibit solid tumors.

## 4. Discussion

Histone acetylation and deacetylation play an important role in the regulation of gene expression in eukaryotic cells [34, 35]. The protein encoded by HDAC1 is part of the histone deacetylase complex. Current drug progresses related to HDAC1 show that HDACi can increase the acetylation degree of intracellular histones and nonhistones, improve the expression level of P21, P53, and other genes, and then achieve the effect of inhibiting tumor cells [36, 37]. This kind of drug can also show a good synergistic therapeutic effect when combined with a variety of chemotherapy drugs and has low toxicity in the effective inhibitory dose range, which has made HDACi a new targeted antitumor drug with wide application. The discovery of new marker targets to establish related gene networks will help diagnose and improve treatment effects.

In previous work, we found that high expression of HDAC1 is a risk factor for LUAD patients, leading to increased mortality. We analyzed transcription and sequencing data from more than 1000 clinical samples from GEO and TCGA databases, confirming that the HDAC1 gene had little difference in copy number in LUAD and significantly higher mRNA and protein expression levels than in normal lung glandular tissue. HDAC1 ranks 2–6% among the genes upregulated in some tissues of LUAD. In the LUAD patient sequencing database in TCGA, the mutation frequency of HDAC1 is 1%. The mutation type is mainly amplification. 18 of 1833 people have mutations in HDAC1. We speculate that there are fewer mutations in the genetic content of chromosome HDAC1 in LUAD tissues, but the highly expressed promoter in cancer tissues participates in assisting the expression of HDAC1, thereby increasing the efficiency of transcription or translation.

In the analysis of HDAC1-related gene network interaction diagram and coexpression diagram, related person diagram, positively correlated gene set heatmap, and negatively correlated gene set heatmap, it can be seen that HDAC1 is positively correlated with MARCKSL1, EIF3I, and other genes. Among them, the MARCKSL1 is related to the occurrence and development of many types of cancers. Relevant literature has proved that MARCKSL1 promotes the progression of LUAD by regulating epithelial-mesenchymal transition (EMT) and can be used as a new therapeutic target for LUAD [38]. EIF3I is a proto-oncogene that is overexpressed in a variety of tumors. It upregulates vascular endothelial growth factor *A*, promotes cell proliferation, and promotes embryonic development and angiogenesis in tumorigenesis [39]. EIF3I is very important for the regulation of protein synthesis, cell proliferation, cell cycle progression, and tumorigenesis [40, 41]. Our results show that HDAC1 has a strong correlation with overexpressed genes in tumors.

In the enrichment analysis of the target gene set, the gene set related to HDAC1 is enriched in multiple GO pathways. Our enrichment analysis results show that the expression of HDAC1 protein is mainly located in the nucleus, its molecular function is mainly protein binding, and the biological process involved is mainly biological regulation. HDAC1 also participates in many molecular signaling pathways on the KEGG pathway. We found that HDAC1 in LUAD is related to the kinase network including CDK and cyclin and the Notch signaling pathway [42, 43]. These kinases are protein complexes that mainly regulate the cell cycle and are closely related to the stable proliferation of cells. Overexpression of HDAC1 can cause abnormal cell cycle regulator E2F transcription factor, which leads to the production of cancer cells [44]. The E2F transcription factor is one of the key regulators of the G1-S phase transition in the cell cycle. Our analysis shows that the E2F transcription factor is an important target of HDAC1, and HDAC1 regulates the proliferation of LUAD cells through the abnormality of this factor.

Regarding the Notch signaling pathway [45], it has been documented that the overexpression of intracellular segments (NICD) in mouse alveolar epithelial cells induces hyperplasia and eventually leads to LUAD. Relevant literature proves that Notch1 is necessary for Kras-induced LUAD and can control tumor cell survival through TP53 [46]. Notch1 and Notch3 signal transduction promotes tumor cell proliferation and inhibits apoptosis in certain NSCLC cell lines [47]. In our results, the overexpression of HDAC1 can lead to the inhibition of the CSL transcription factor, thereby inhibiting the transcription of related genes, inhibiting the activation of the Notch signaling pathway, and causing the Notch signaling pathway to be abnormal. The target genes of the Notch signal are mostly basic helix-loop-helix transcription factors, such as nuclear factor-*κ*B (NF-*κ*B), cyclin D1, c-myc gene, P21 gene, P27 gene, AKT serine/threonine kinase, and the mechanistic target of rapamycin (mTOR). The target genes of the Notch signaling pathway have been well documented in the occurrence and progression of tumors. They regulate the transcription of other genes directly related to cell differentiation. In the relevant literature, abnormal regulation of the Notch pathway may occur through a variety of mechanisms, including mutation activation or inactivation, overexpression, posttranslational modification, and epigenetic regulation. More and more evidence shows that Notch1 is a putative oncogene in LUAD.

In human non-small-cell lung cancer, the gain-of-function mutation of the Notch1 gene and the weakening of the pathway due to the loss of NUMB have been described. Relevant literature shows that the Notch signaling pathway has a cancer-promoting effect on lung cancer. The Notch signaling pathway interacts with multiple signaling pathways, including Wnt, TGF-*β*, and HER-2. We suspect that the overexpression of HDAC1 causes the transcriptional inhibition of Notch signal target genes to inhibit the progression of LUAD. This study provides relevant evidence for the correlation of HDAC1 overexpression in the occurrence and progression of LUAD and its potential as a marker for LUAD. They include HDAC1-interacting genes such as EIF3I, MARCKSL1, and kinase network genes including cyclin-dependent kinase (CDK). In addition, the Cyclin and Notch signaling pathways involved in HDAC1 are closely related to cell growth, development, differentiation, and diffusion. HDAC1 is particularly related to several tumor-related kinases (such as CDK), transcription factors (EIF3I, CSL, E2F), and signaling pathways (NOTCH).

In this study, we used online tools based on the most popular bioinformatics theory to analyze target genes from the tumor data in the public database. Compared with traditional chip screening, this method has the advantages of a large sample size, low cost, and simple operation. At the same time, the TCGA database also has some limitations. First, there is no sufficient data on the LUAD sample in TCGA. Only three ethnic groups were included in the LUAD sample, and stage 4 patients were relatively rare. The absence and insufficiency of data directly affect our analysis. Another limitation is that transcriptome sequencing can only detect static mutations; it cannot directly provide information about the level of protein activity or expression. These issues should be addressed in subsequent activities using molecular biology techniques. Our results suggest that HDAC1 may be used as a prognostic marker for cancer treatment in future clinical practice. At the same time, due to its important role in tumor genesis and development, this study provides a theoretical basis for the HDAC1 target therapy of LUAD.

## 5. Conclusion

With the understanding of the related role of HDAC1 in cells, we concluded from the results of HDAC1 expression in LUAD, mutation frequency and form, gene interaction, and functional enrichment analysis that the abnormal transcription factors and related signal pathway changes caused by HDAC1 overexpression are related to the occurrence and development of LUAD. There are limitations in the process of our study, which need to be improved through further biochemical experiments in subsequent activities. Our study helps uncover gene networks associated with LUAD and new therapeutic targets.

## Data Availability

All data generated or analyzed during this study are included in this published article.

## Supplementary Materials

Supplementary Materials Table S1. The latest clinical drugs for HDAC1.
