# Supplementary material for: Histone Deacetylase 1 Expression and Regulatory Network in Lung Adenocarcinoma Based on Data Mining and Implications for Targeted Treatment
Source: J Oncol. 2023 Jan 4;2023:2745074. doi: 10.1155/2023/2745074 (PMC9833904; doi:10.1155/2023/2745074)
Supplement: Supplementary Materials — Table S1. The latest clinical drugs for HDAC1. [file 2745074.f1.docx]

Table S1: The latest clinical drugs for HDAC1.

| **Search term** | **Match term** | **Match type** | **Gene** | **Drug** | **Interaction types** | **Sources** | **Pmids** |
| --- | --- | --- | --- | --- | --- | --- | --- |
| **HDAC1** | **HDAC1** | **Definite** | **HDAC1** | **CHEMBL152543** | **inhibitor** | **TTD** |  |
| **HDAC1** | **HDAC1** | **Definite** | **HDAC1** | **VORINOSTAT** | **Inhibitor** | **TALC\|MyCancerGenome\|TdgClinicalTrial\|GuideToPharmacologyInteractions\|ChemblInteractions\|TEND\|DrugBank\|TTD** | **17694093\|11752352** |
| **HDAC1** | **HDAC1** | **Definite** | **HDAC1** | **ABEXINOSTAT** | **Inhibitor** | **TALC \| Tdg Clinical Trial\| Guide To Pharmacology Interactions \|Drug Bank** |  |
| **HDAC1** | **HDAC1** | **Definite** | **HDAC1** | **APICIDIN** | **Inhibitor** | **Guide To Pharmacology Interactions** |  |
| **HDAC1** | **HDAC1** | **Definite** | **HDAC1** | **BELINOSTAT** | **Inhibitor** | **TALC\|MyCancerGenome\|TdgClinicalTrial\|GuideToPharmacologyInteractions\|ChemblInteractions\|DrugBank\|TTD** |  |
| **HDAC1** | **HDAC1** | **Definite** | **HDAC1** | **BUTANOIC ACID** | **Inhibitor** | **Guide to Pharmacology Interactions** |  |
| **HDAC1** | **HDAC1** | **Definite** | **HDAC1** | **CHEMBL1801250** | **Inhibitor** | **TALC \|Guide to Pharmacology Interactions** |  |
| **HDAC1** | **HDAC1** | **Definite** | **HDAC1** | **CUDC-101** | **Inhibitor** | **TALC\|TdgClinicalTrial\|GuideToPharmacologyInteractions\|ChemblInteractions** |  |
| **HDAC1** | **HDAC1** | **Definite** | **HDAC1** | **CUDC-907** | **Inhibitor** | **Guide to Pharmacology Interactions \| Chembl Interactions** |  |
| **HDAC1** | **HDAC1** | **Definite** | **HDAC1** | **DACINOSTAT** | **Inhibitor** | **Guide to Pharmacology Interactions \|TTD** |  |
| **HDAC1** | **HDAC1** | **Definite** | **HDAC1** | **ENTINOSTAT** | **Inhibitor** | **TALC\|TdgClinicalTrial\|GuideToPharmacologyInteractions\|ChemblInteractions\|TTD** |  |
| **HDAC1** | **HDAC1** | **Definite** | **HDAC1** | **CHEMBL1213492** | **Inhibitor** | **TALC \| Tdg Clinical Trial \| Guide to Pharmacology Interactions \|TTD** |  |
| **HDAC1** | **HDAC1** | **Definite** | **HDAC1** | **MOCETINOSTAT** | **Inhibitor** | **TdgClinicalTrial\|GuideToPharmacologyInteractions\|ChemblInteractions\|DrugBank\|TTD** | **17455259\|17868033** |
| **HDAC1** | **HDAC1** | **Definite** | **HDAC1** | **NEXTURASTAT A** | **Inhibitor** | **Guide to Pharmacology Interactions** |  |
| **HDAC1** | **HDAC1** | **Definite** | **HDAC1** | **PANOBINOSTAT** | **Inhibitor** | **TALC\|MyCancerGenome\|TdgClinicalTrial\|GuideToPharmacologyInteractions\|DrugBank\|CancerCommons\|TTD** | **17145876\|17455259** |
| **HDAC1** | **HDAC1** | **Definite** | **HDAC1** | **CHEMBL1851943** | **Inhibitor** | **TALC \| TdgClinicalTrial\|GuideToPharmacologyInteractions\|DrugBank\|CancerCommons** |  |
| **HDAC1** | **HDAC1** | **Definite** | **HDAC1** | **QUISINOSTAT** | **Inhibitor** | **Guide to Pharmacology Interactions** |  |
| **HDAC1** | **HDAC1** | **Definite** | **HDAC1** | **RICOLINOSTAT** | **Inhibitor** | **Guide to Pharmacology Interactions** |  |
| **HDAC1** | **HDAC1** | **Definite** | **HDAC1** | **ROMIDEPSIN** | **Antagonist\| inhibitor** | **TALC \| My Cancer Genome \|Tdg Clinical Trial \| Guide To Pharmacology Interactions \| Chemb l Interactions \| TEND \| Drug Bank** | **21587264** |
| **HDAC1** | **HDAC1** | **Definite** | **HDAC1** | **SCRIPTAID** | **Inhibitor** | **Guide to Pharmacology Interactions \| TTD** |  |
| **HDAC1** | **HDAC1** | **Definite** | **HDAC1** | **TACEDINALINE** | **Inhibitor** | **Guide to Pharmacology Interactions \| Chemb l Interactions \|TTD** |  |
| **HDAC1** | **HDAC1** | **Definite** | **HDAC1** | **TRICHOSTATIN** | **Inhibitor** | **Guide to Pharmacology Interactions** |  |
| **HDAC1** | **HDAC1** | **Definite** | **HDAC1** | **TUCIDINOSTAT** | **Inhibitor** | **Guide to Pharmacology Interactions** |  |
| **HDAC1** | **HDAC1** | **Definite** | **HDAC1** | **VALPROIC ACID** | **Inhibitor** | **TALC \| Guide to Pharmacology Interactions** |  |
| **HDAC1** | **HDAC1** | **Definite** | **HDAC1** | **PHENYLBUTANOIC ACID** | **Inhibitor** | **TTD** |  |
| **HDAC1** | **HDAC1** | **Definite** | **HDAC1** | **SODIUM BUTYRATE** | **Inhibitor** | **TTD** |  |
| **HDAC1** | **HDAC1** | **Definite** | **HDAC1** | **CHEMBL100014** | **Inhibitor** | **TALC \| TTD** |  |
| **HDAC1** | **HDAC1** | **Definite** | **HDAC1** | **CHEMBL191091** | **Inhibitor** | **TTD** |  |
| **HDAC1** | **HDAC1** | **Definite** | **HDAC1** | **PYROXAMIDE** | **Inhibitor** | **TTD** |  |
| **HDAC1** | **HDAC1** | **Definite** | **HDAC1** | **CHEMBL152133** | **Inhibitor** | **TTD** |  |
| **HDAC1** | **HDAC1** | **Definite** | **HDAC1** | **CHEMBL491491** | **Inhibitor** | **TTD** |  |
| **HDAC1** | **HDAC1** | **Definite** | **HDAC1** | **CHLAMYDOCIN** | **Inhibitor** | **TTD** |  |
| **HDAC1** | **HDAC1** | **Definite** | **HDAC1** | **DEPUDECIN** | **Inhibitor** | **TTD** |  |
| **HDAC1** | **HDAC1** | **Definite** | **HDAC1** | **CHEMBL491316** | **Inhibitor** | **TTD** |  |
| **HDAC1** | **HDAC1** | **Definite** | **HDAC1** | **SODIUM PHENYLBUTYRATE** | **Inhibitor** | **TALC** |  |
| **HDAC1** | **HDAC1** | **Definite** | **HDAC1** | **CHEMBL191482** |  | **Tdg Clinical Trial** |  |
| **HDAC1** | **HDAC1** | **Definite** | **HDAC1** | **PANOBINOSTAT LACTATE** | **Inhibitor** | **Chemb l lnteractions** |  |
| **HDAC1** | **HDAC1** | **Definite** | **HDAC1** | **OXAMFLATIN** | **Inhibitor** | **TTD** |  |
